# Synchronous multiple small bowel intussusceptions in an adult with blue rubber bleb naevus syndrome: Report of a case and review of literature

**DOI:** 10.1186/1749-7922-3-3

**Published:** 2008-01-18

**Authors:** Clement Lee, Debasish Debnath, Tara Whitburn, Mark Farrugia, Federico Gonzalez

**Affiliations:** 1 Department of Surgery, Newham University Hospital, Plaistow, London, E13 8SL, UK; 2 Department of Radiology, Newham University Hospital, Plaistow, London, E13 8SL, UK

## Abstract

**Background:**

Blue rubber bleb naevus syndrome (BRBNS), is an uncommon condition characterised by cavernous haemangiomas of skin and gastrointestinal tract. The most common complication of this syndrome is gastrointestinal bleeding. Intussusception of bowel, although a known complication, has rarely been reported.

**Case presentation:**

We report the case of a 37-year-old man who presented with multiple intussusceptions of small bowel. He required an urgent laparotomy and bowel resections. He suffered from BRBNS. This is the first reported case of multiple synchronous intussusceptions affecting both jejunum and ileum, secondary to haemangiomas occurring in an adult with BRBNS. The underlying conditions of acute abdomen in patients with BRBNS may include intramural haemorrhage, infarction, volvulus or intussusception of bowel. Treatment options include pharmacological manipulation, bowel resection, and interventions such as sclerotherapy, angiographic embolisation, endoscopic ligation, electrocautery and laser photocoagulation for visceral lesions.

**Conclusion:**

A high index of suspicion is required whilst dealing with acute abdomen in patients with BRBNS. Clinical trials may provide some answers as to the preference of treatment in individual cases, as the current level of evidence does not offer a clear choice of optimal treatment.

## Background

Management of acute abdomen in a patient suffering from blue rubber bleb naevus syndrome can be challenging. We report a unique case of intestinal intussusception in such a patient, who was treated successfully without any complication. We discuss relevant details and provide a review of literature. Optimal management of this condition remains unclear. We intend to make readers aware of potentially serious underlying conditions while dealing with acute abdomen in patients of blue rubber bleb naevus syndrome and highlight the need for optimising various treatment options.

## Case presentation

A 37-year-old male presented at Accident and Emergency department with chief complaints of colicky abdominal pain, nausea and vomiting of twelve hours' duration. The pain was generalised in nature, moderate in severity, and not associated with any aggravating or relieving factor. There was no alteration of bowel habit. His past medical history included multiple blood transfusions for 'anaemia' since childhood, Hepatitis C and blue rubber bleb naevus syndrome (BRBNS). The latter was diagnosed following 'bowel surgery for internal bleeding' seven years ago at a different hospital. He had been treated with endoscopy-guided argon plasma coagulation and interferon α therapy to induce regression of gastrointestinal haemangiomas with limited success. No other family member had suffered from a similar condition.

**Figure 1:**
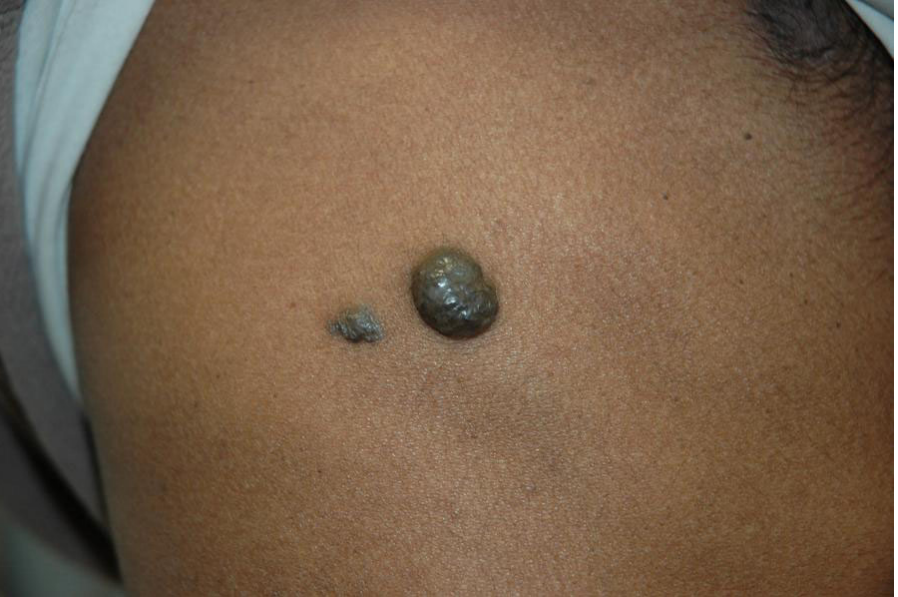
Cutaneous haemangiomas affecting right chest wall.

**Figure 2:**
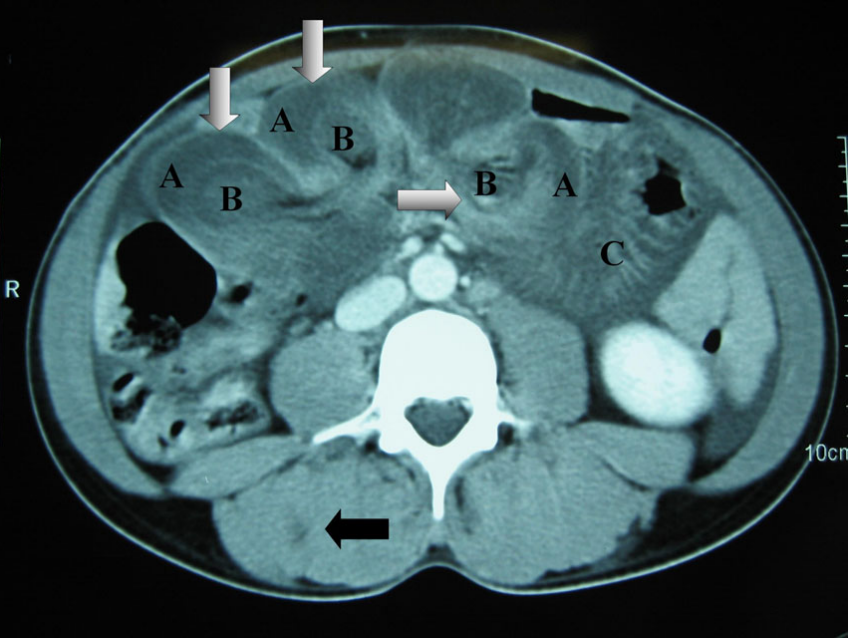
CT scan of abdomen showing i) multiple intussusceptions demonstrating doughnut signs (white arrows), intussusceptiens [A], intussusceptum [B], distended loop of small bowel [C] and ii) haemangioma of right quadratus lumborum muscle (dark arrow).

On admission the vital observations were stable. General examination showed several subcutaneous compressible bluish skin lesions around neck, chest and abdomen (Figure 1). Abdominal examination revealed soft and mildly distended abdomen with moderate epigastric tenderness. The bowel sounds and digital rectal examination were normal. Laboratory investigations showed haemoglobin 91 g/l, WBC 4.8 × 10^9^/l, platelet 144 × 10^9^/l. Urea, creatinine, electrolytes, liver function tests, amylase and coagulation screen were within normal limits. Plain X-ray of chest was normal. Abdominal radiograph showed distended loops of small bowel. Computed Tomography (CT) of abdomen revealed i)dilated loops of small bowel ii)'doughnut' signs, suggestive of intussusception, affecting small bowel at three different levels iii)subcapsular hepatic haemangiomas affecting segment six and iv)intramuscular haemangioma involving right quadratus lumborum muscle (Figure 2).

**Figure 3:**
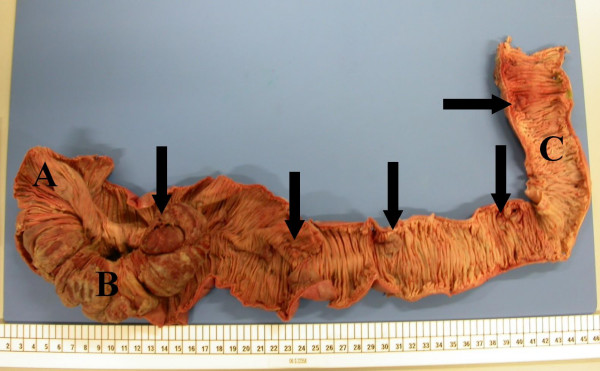
Specimen of limited right hemicolectomy showing terminal ileum [A], caecum [B] and ascending colon [C], and demonstrating multiple haemangiomas affecting mucosa and submucosa (arrows).

He underwent laparotomy that revealed three irreducible small bowel intussusceptions at the levels of proximal jejunum, mid-ileum and distal ileum. Each of the intussusception was led by a large intraluminal haemangioma. There was also a localised perforation at mid-ileal level. Resections of the intussusceptions with primary anastomosis of small bowel segments and limited right hemicolectomy were performed (Figure 3). The length of the remaining small bowel was 150 cm. Histology of the specimens confirmed intussusceptions of small bowel with gangrenous changes. Multiple haemangiomas involving serosa and submucosa were also noted. There was no feature of dysplasia or malignancy.

The postoperative course and follow-ups up to 6 months were uneventful. Further follow-ups were planned with gastroscopy, video capsule enteroscopy and colonoscopy.

## Discussion

Blue rubber bleb naevus syndrome, first coined by Bean in 1958, is an uncommon entity characterised by multiple blue naevi affecting skin and gastrointestinal organs [1]. The skin lesions appear at birth or in childhood. They are mostly located on the upper extremities and trunk [2]. In the case described, the naevi were mostly distributed around the trunk. Gastrointestinal lesions occur mostly in the small intestine and bleed easily, which results in iron-deficiency anaemia [2]. Our patient had widespread lesions affecting stomach, small bowel and colon. Such lesions can give rise to intramural haemorrhage, infarction, volvulus and intussusception [3]. Intussusception of bowel in an adult without history of abdominal surgery often warrants prompt surgical intervention that usually entails bowel resection without reduction, whenever possible [4]. A high index of suspicion is warranted while dealing with acute abdomen in patients with known BRBNS or typical skin lesions that are suggestive of BRBNS. In deed, the patient reported in the case had multiple synchronous intussusceptions of small bowel affecting both jejunum and ileum, a reflection of the extent of visceral involvement. Unrestricted literature search of Medline till February 2007 showed that such a case had never been reported before. One report in the literature, closest to our case, entailed a 22-year-old man, who also had multiple intussusceptions of iluem but the jejunum was spared, making our case report unique [5].

Haemangiomatous lesions in BRBNS have also been noted to occur in the central nervous system, eye, nasopharynx, oropharynx, parotid glands, liver, spleen, pleura, lung, trachea, skeletal muscle, penis, vulva, urinary bladder, pericardium and bone [2,5-9]. There have been reported associations of BRBNS with disseminated intravascular coagulopathy, pulmonary hypertension, deep vein thrombosis, cerebellar medulloblastoma, hypernephroma, chronic lymphocytic leukaemia and angiokeratoma [10]. Differential diagnoses of BRBNS include Rendu-Osler-Weber, Klippel-Trenaunay, Maffucci, Sturge-Weber, Von- Hippel-Lindau and Cobb's Syndrome [11]. Small intestinal malignant melanoma may also present as anaemia, gastrointestinal bleed and abdominal pain. Diagnosis of such lesions can be difficult, is frequently delayed and made when complications occur [12]. Differentiation between melanoma and haemangiomatous lesions of BRBNS can be very difficult and final diagnosis often rests on histology. Only one case had been reported in the literature, citing an incidental malignant melanoma in association with progressive BRBNS [13].

BRBNS is mostly sporadic in nature [14]. However, autosomal dominance and linkage to 9p chromosome have been documented [15]. Mutation affecting tyrosine kinase (TIE2) receptor, a participant in the angiogenesis pathway, has been implicated in certain cases [16]. The patient under discussion had no family history of this syndrome, suggesting a de novo mutation as the possible underlying aetiology. Histological descriptions of naevi range from arteriovenous malformation and cavernous haemangioma to capillary telangiectasia [10]. Such lesions have not reported to be associated with malignant changes [5].

Endoscopy remains the common method of detecting gastrointestinal lesions. Abdominal radiographs may show calcifications representing well organised thrombosed visceral lesions. Barium studies may show filling defects caused by wide based sessile polyps [17]. CT and magnetic resonance imaging may aid in localisation of the lesions [18,19]. Angiography may reveal small haemangiomatous lesions and is most useful in the case of active bleeding [17].

Cosmetically compromising skin lesions can be treated by laser surgery or excision. Treatment options of gastrointestinal lesions include i) sclerotherapy, ii) angiographic embolisation, ii) endoscopic (sometimes assisted by enterotomy) ligation, electrocautery and laser photocoagulation [20,21]. Bowel resection is reserved for localised lesions, significant bleeding that cannot be controlled suitably by endoscopy or radiology, and for complications such as ischaemia and intussusception [21]. Role of elective radical surgical excision remains debatable [22,23]. Anti-angiogenic agents including corticosteroids, interferon α therapy and gamma globulin have been attempted [24,25]. However, the lesions often returned to their pre-treatment levels soon after the treatment was discontinued [24]. Our patient received interferon α therapy in the past without any lasting effect. None of these methods has demonstrated any convincing evidence of durable success. Choice of optimal treatment in individual cases remains unclear. Comparative trials need to be performed in order to assess the long term success prior to considering any of these therapeutic approaches as the treatment of choice. However infrequent occurrence of BRBNS could be a limiting factor in organising such trials.

## Conclusion

We describe a unique case of blue rubber bleb naevus syndrome, a rare disease manifested by distinctive cutaneous and gastrointestinal haemangiomatous lesions. The commonest presentation is iron deficiency anaemia. The cutaneous lesions may serve as warning signs of possible intestinal intussusception, intramural haemorrhage, infarction or volvulus in patients presenting with acute abdomen. Many therapeutic options are available, but no single therapy has yet been established as the treatment of choice. Future research involving well designed trials may shed light in this area.

## Abbreviations

BRBNS – Blue rubber bleb naevus syndrome

CT – Computed tomography

## Competing interests

The author(s) declare that they have no competing interests.

## Authors' contributions

CL: Involved in peri-operative care including all investigations, designed the case report, collated the information, searched literature and drafted the manuscript.

DD: Involved in peri-operative care including all investigations, designed the case report, collated the information, searched literature and drafted the manuscript.

TW: Involved in peri-operative care and all investigations, assisted in providing a critical appraisal of the manuscript.

MF: Made the radiological diagnoses and assisted in preparing radiology image, advised on the format and design of the manuscript.

FG: Performed the surgery, involved in all investigations, assisted in literature search, writing and editing of the manuscript.

All authors have reviewed and approved the final manuscript.
